# *Porcisia* transmission by prediuresis of sand flies

**DOI:** 10.3389/fcimb.2022.981071

**Published:** 2022-08-10

**Authors:** Jovana Sadlova, Dominika Bacikova, Tomas Becvar, Barbora Vojtkova, Marion England, Jeffrey Shaw, Petr Volf

**Affiliations:** ^1^ Department of Parasitology, Faculty of Science, Charles University, Prague, Czechia; ^2^ Transmission Biology, The Pirbright Institute, Surrey, United Kingdom; ^3^ Departamento de Parasitologia, Instituto de Ciências Biomédicas, Cidade Universitária, São Paulo, Brazil

**Keywords:** *Porcisia deanei*, *Porcisia hertigi*, *Lutzomyia*, *Culicoides*, prediuresis, Malpighian tubules, contaminative transmission

## Abstract

Parasites of the genus *Porcisia*, together with the genus *Endotrypanum*, form a sister clade to the species-rich and medically important genus *Leishmania*. Both *Porcisia* species, *P. hertigi* and *P. deanei*, are dixenous parasites of Neotropical porcupines. Almost 50 years after their first discovery, knowledge of their life cycle remains poor and their insect vectors are unknown. Because competent vectors of their closest phylogenetic relatives, genera *Endotrypanum* and *Leishmania*, are phlebotomine sand flies (Diptera: Psychodidae) and/or biting midges (Diptera: Ceratopogonidae), we examined here the potential of both sand flies and biting midges to transmit *Porcisia* parasites. The insects (*Lutzomyia longipalpis, L. migonei* and *Culicoides sonorensis*) were exposed to parasites through the chicken skin membrane and dissected at various time intervals post bloodmeal. Potentially infected females were also allowed to feed on the ears of anaesthetized BALB/c mice and the presence of parasite DNA was subsequently confirmed in the mice by PCR. *Porcisia hertigi* did not survive defecation in *L. longipalpis* or *L. migonei*, suggesting that these sand fly species are unlikely to serve as natural vectors of this parasite. Similarly, *P. hertigi* infections were lost in *Culicoides* midges. In contrast, mature *P. deanei* infections developed in 51-61% of *L. longipalpis* females, 7.3% of *L. migonei* females and 7.7% of *Culicoides sonorensis* females. In all three vector species, *P. deanei* colonized predominantly Malpighian tubules and produced metacyclic infective forms. Transmission of *P. daenei* to BALB/c mice was demonstrated *via* the prediuresis of *L. longipalpis* females. This mode of transmission, as well the colonization of Malpighian tubules as the dominant tissue of the vector, is unique among trypanosomatids. In conclusion, we demonstrated the vector competence of *L. longipalpis* for *P. deanei* but not for *P. hertigi*, and further studies are needed to evaluate competence of other Neotropical vectors for these neglected parasites.

## Introduction

*Porcisia* are dixenous parasites of Neotropical porcupines. The genus was established in 2016 for two species, originally described as *Leishmania hertigi* and *L. deanei* (Espinosa et al., 2016). These two species are found in different porcupine species, in different geographical regions, and are clearly separated by molecular markers (Espinosa et al., 2016).

*Leishmania hertigi* was originally found in *Coendou rothschildi* (according to the taxonomical revision of the genus *Coendou* (Voss, 2015) the species is now called *Coendou quichua*) from Central Panama (Herrer, 1971) and later in *Coendou mexicanus* from Costa Rica (Zeledón et al., 1977). While the parasite was widespread in Panama and showed very high prevalence rates (75 - 100%), it was not so common in Costa Rica, where it was detected in a single individual out of 25 porcupines captured in three different localities (Herrer, 1971; Zeledón et al., 1977). Parasites are harmless to the mammalian host. Although they spread in the skin throughout the body and often enter internal organs, they do not cause any gross skin alteration (Herrer, 1971). It also appears to be host-specific, despite extensive searches, it has not been detected in hosts other than *Coendou* (Herrer et al., 1966) and laboratory infections of *Mesocricetus auratus*, *Sigmodon hispidus*, *Jaculus jaculus* and *Cavia porcellus* have been short-lasting and limited to the inoculation site (Herrer, 1971).

Similar parasites of porcupines (*Coendou prehensilis, C. nycthemera* and *Coendou* sp.) have been reported from Brazil (Deane et al., 1974; Lainson and Shaw, 1977) and named first *Leishmania hertigi deanei* (Lainson and Shaw, 1977), later raised to the specific status of *L. deanei* by Lainson and Shaw (1987). Parasites later described in Brazil (da Silva et al., 2013; Thies et al., 2018) as *L. hertigi* are probably also *L. deanei*. Their species identity had been based on 18S rDNA sequences, which are conserved and may not distinguish between these closely related species (Espinosa et al., 2016). Similar to *L. hertigi*, *L. deanei* did not produce any visible pathological effects in its natural host and attempts to infect hamsters and guinea-pigs were unsuccessful (Lainson and Shaw, 1977).

Almost 50 years after their first discovery; the vectors of both *Porcisia* species remain unknown. Since *Porcisia* are closely related to genera *Endotrypanum* and *Leishmania* (Espinosa et al., 2016), they are most likely transmitted by sand flies of the family Psychodidae (proven vectors of most species of the genus *Leishmania* and likely vectors of the genus *Endotrypanum;* Franco et al., 1997; Maroli et al., 2013) or biting midges of the family Ceratopogonidae (recently proven to be competent vectors of the *Leishmania* (*Mundinia*) subgenus; Dougall et al., 2011; Chanmol et al., 2019; Becvar et al., 2021). Experimental studies from the 1960s-1970s described the poor development of *P. hertigi* and *P. deanei* in *L. longipalpis* (Lainson et al., 1977; Croft and Molyneux, 1979) and *L. sanquinaria* (Herrer et al., 1966). In Sinop, Brazil, *Porcisia* DNA was recently detected in *Lutzomyia antunesi* (Thies et al., 2018). However, whether the parasite is able to complete development in this *Lutzomyia* species is unclear.

**Figure 1:**
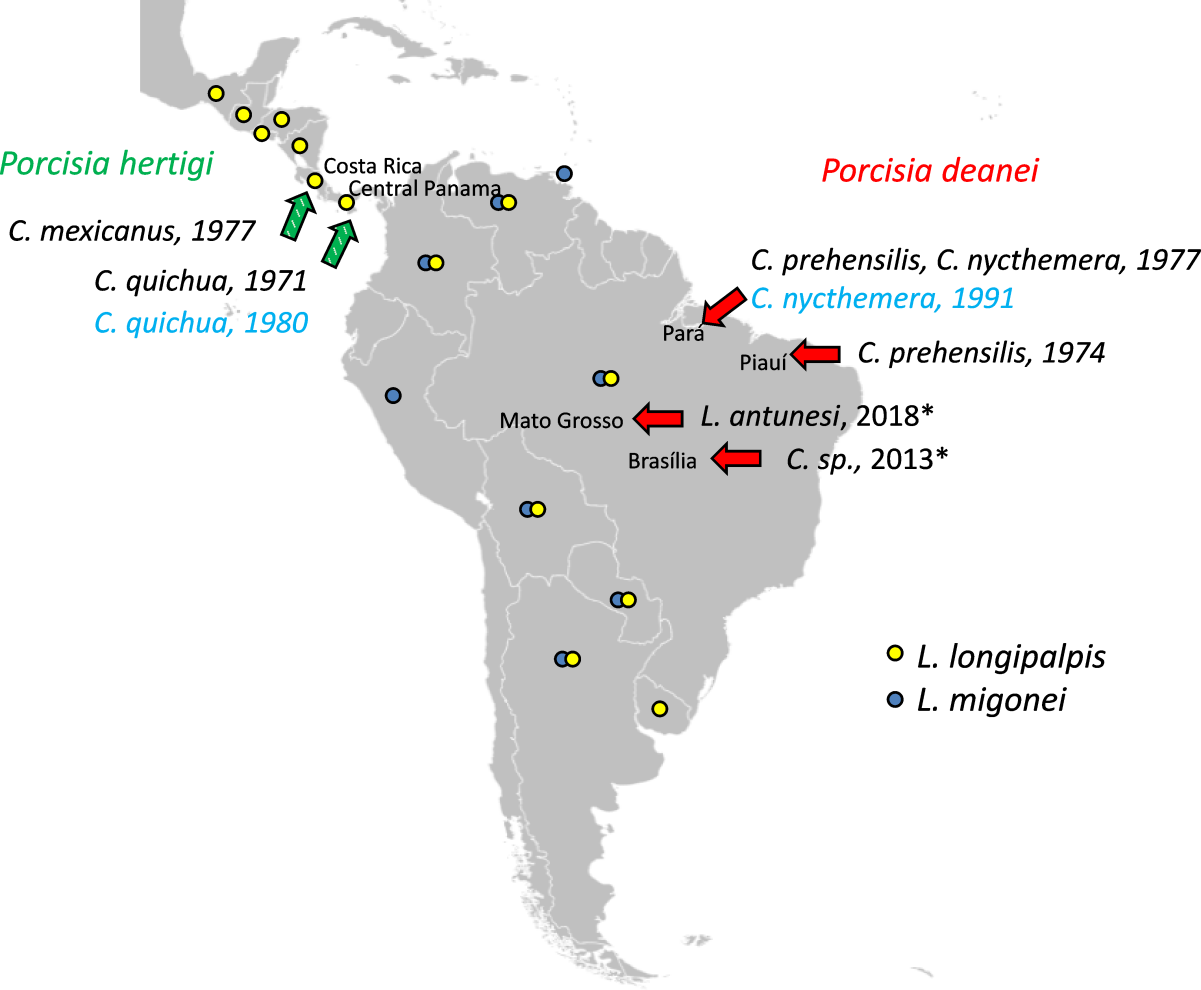
Locations of *Porcisia* reports. Green arrows indicate *P. hertigi* records and red arrows *P. deanei* records with publication/isolation dates and host species, based on (Herrer, 1971; Deane et al., 1974; Zeledón et al., 1977; Lainson and Shaw, 1977; da Silva et al., 2013). Asterisks indicate two *Porcisia* records identified as *P. hertigi*, but the species identification method was not sufficient to distinguish the two *Porcisia* species, and based on geographic region and host species, both are *P. deanei* (Espinosa et al., 2016). Strains used in this study are indicated in blue. Countries with records of *L. longipalpis* and *L. migonei* according to Shimabukuro et al., 2017 are marked with yellow and blue circles, respectively. The blank map source – https://commons.wikimedia.org.

Our study was aimed at detailed assessment of the vector competence of two Neotropical sand fly species, *L. longipalpis* and *L. migonei*, known as vectors of *Leishmania infantum* and *Leishmania braziliensis* (Guimarães et al., 2016; Dvořák et al., 2018; Alexandre et al., 2020). According to Shimabukuro et al. (2017), they share a distribution range with *P. deanei* and *L. longipalpis* also with *P. hertigi* (**Figure 1**). In addition, we tested development of both *Porcisia* species in *Culicoides sonorensis*, North American biting midge known to experimentally transmit *L. (Mundinia)* sp. (Chanmol et al., 2019; Becvar et al., 2021).

## Materials and methods

### Sand flies, parasites and mice

Colonies of sand flies *L. longipalpis* and *L. migonei* (both originally from Brazil) were maintained in the insectary of the Department of Parasitology, Charles University in Prague, under standardized conditions (26°C, fed on 50% sucrose and photoperiod 14 h light/10 h dark) as described previously (Volf and Volfova, 2011). *Culicoides sonorensis* biting midges were sent to Charles University from The Pirbright Institute, UK and kept at the same conditions as the sand flies.

Promastigotes of *Porcisia deanei* (MCOE/BR/91/M13541; TCC 258) isolated from *C. nycthemera* in Acara region, Brazil, *P. hertigi* (MCOE/PA/80/C8; TCC 260) isolated from *C. quichua* in 1980 in central Panama and *L. infantum* (MHOM/TR/2000/OG-VL) were cultured in M199 medium (Sigma) containing 10% heat-inactivated foetal calf serum (FBS, Gibco) and 2% sterile human urine (provided by J.Sa.) supplemented with 1% BME vitamins (Basal Medium Eagle, Sigma) and 250 µl/mL amikacin (Amikin, Bristol-Myers Squibb).

BALB/c mice originated from AnLab. s.r.o. (Harlan Laboratories, USA, Nederland). Animals were housed in T3 breeding containers (Velaz) equipped with bedding (German Horse Span Pferde) and breeding material (Woodwool), receiving standard ST-1 feed (Velaz) and water ad libitum, with a 12 h light/12 h dark photoperiod, temperature 22–25°C and humidity 40–60%.

### Experimental infections of sand flies and biting midges

Promastigotes from log-phase cultures were resuspended in heat-inactivated defibrinated ram blood at a concentration of 1 × 10^6^ promastigotes/ml. Female insects were infected by feeding through a chick-skin membrane and engorged individuals were maintained in the same conditions as the colony for subsequent dissections at different time intervals. Evaluation of infections was difficult in freshly engorged females (day 1 PBM): while the presence of control *L. infantum* was evident under the light microscope, both *Porcisia* species were indistinguishable from red blood cells due to their small rounded body and very short or absent flagella. Therefore, the infection rate at this time point was estimated from Giemsa-stained gut smears and infection intensity was scored as moderate. The intensity and localisation of infection in later time intervals were evaluated under the light microscope; the infections were scored as light (<100 parasites per gut), moderate (100-1000 parasites per gut) or heavy (>1000 parasites per gut) (Myskova et al., 2008) Differences in infection intensity were tested by Chi-Square test using SPSS Version 27.

### Morphological analysis of parasites

Morphology of parasites from infected guts was evaluated from methanol-fixed and Giemsa-stained gut smears. The stained gut smears were also used to assess the infection rate of *Porcisia* on day 1 PBM. Promastigotes were examined by light microscopy with an oil immersion objective and photographed using the Olympus DP70 camera. Body length, body width and flagellar length of parasites were measured using Image J software. Promastigotes were scored as procyclic forms (present in the bloodmeal) when flagellum length ≤ body length, metacyclic forms when flagellum length ≥ 2 times body length, leptomonad forms when flagellum length < 2 times body length and body length was < 14 µm and < 8 µm in *Leishmania* and *Porcisia*, respectively, and elongated nectomonads when flagellum length < 2 times body length and body length ≥ 14 µm and ≥ 8 µm in *Leishmania* and *Porcisia*, respectively, according to (Sadlova et al., 2010). In *Leishmania* species, a threshold of 14 µm is used to distinguish elongated nectomonads from leptomonads (Sadlova et al., 2010). However, this threshold cannot be used for the smaller promastigotes of the genus *Porcisia*, so we set a threshold of 8 µm by comparing measurements of the two genera (**Supplementary File 1**). Haptomonads characterized by a disc-shaped extension of the flagellar tip were recorded but not quantified because these forms, attached to the sand fly gut, are underscored on gut smears.

### Examination of urine droplets

Female *Lutzomyia longipalpis*, 10 - 14 days after infective bloodmeal, were individually forced to feed on microcapillaries containing M199 medium according to (Hertig and Mcconnell, 1963). During feeding, droplets excreted from abdomen were captured on a cover - slip and immediately after feeding, engorged flies were dissected to detect *Porcisia* infections. Droplets from infected flies were air dried, fixed with methanol and stained with Giemsa. The presence of parasites was examined by light microscopy using an oil immersion objective and their morphology was evaluated as described above.

### Transmission experiments and PCR detection of parasites

Experimentally infected insects were maintained for 10 days at 26°C and then allowed to feed on BALB/c mouse anaesthetized with a mixture of ketamin and xylazine (62 mg/kg and 25 mg/kg). The female insects were placed in small plastic tubules covered with fine mesh and the tubules were held on the pinnae of the anaesthetized mouse for one hour. Mice were sacrificed immediately or 4 days after the experiment by cervical dislocation under anesthesia. The pinnae (biting site) were excised and stored at -20 °C for PCR. In a single mouse dissected 4 days post exposure, ears draining lymph nodes were also collected. Because *Porcisia* spp. are strongly adapted to porcupines and do not survive for long periods of time in other model hosts (Herrer, 1971), infections could not be monitored over the long term. Insects were dissected immediately post bloodmeal and checked for the presence of parasites under a light microscope. DNA extraction from animal tissues was performed using the High Pure PCR Template Preparation Kit (Roche Diagnostics, Indianapolis, IN) according to the manufacturer’s instructions. The total DNA was used as a template for a nested PCR amplification with the outer primers amplifying 332 bp long 18S sequence (forward primer 18SN1F 5´- GGA TAA CAA AGG AGC AGC CTC TA-3´ and reverse primer 18SN1R 5´– CTC CAC ACT TTG GTT CTT GAT TGA-3´) and inner primers amplifying 226 bp long 18S sequence (forward primer 18SN2F 5′-AGA TTA TGG AGC TGT GCG ACA A-3′ and reverse primer 18SN2R 5′-TAG TTC GTC TTG GTG CGG TC-3′). Samples were subsequently analysed using 1% agarose gel. Reaction mixtures and cycling conditions were as follows:

1. Step of PCR: 3 µl of genomic DNA, 0,5 µl forward primer 18SN1F (10 µM), 0,5 µl reverse primer 18SN1R (10 µM), 10 µl of 2x EmeraldAmp^®^ GT PCR Master Mix (Takara Bio), 6 µl of ddH_2_O.

step 1, 94 °C for 3 min 30 s; step 2, 94 °C for 30 s; step 3, 60 °C for 30 s; step 4, 72 °C for 25 s; step 5, 72 °C for 7 min; followed by cooling at 12 °C. Steps 2–4 were repeated 35 times.

2. Step of PCR: 1 µl of 1. step PCR reaction, 0,5 µl forward primer 18SN2F (10 µM), 0,5 µl reverse primer 18SN2R (10 µM), 10 µl 2x EmeraldAmp^®^ GT PCR Master Mix (Takara Bio), 8 µl ddH_2_O

### Animal experimentation guidelines

The mice were kept and handled in the animal facility of Charles University in Prague in accordance with institutional guidelines and Czech legislation (Act No. 246/1992 and 359/2012 coll. on Protection of Animals against Cruelty in present statutes at large), which is in compliance with all relevant European Union and international guidelines for experimental animals. All experiments were approved by the Committee on the Ethics of Laboratory Experiments of Charles University in Prague and were performed under permission no. MSMT-7831/2020-3 of the Ministry of Education, Youth and Sports. The investigators are certificated for animal experiments by the Ministry of Agriculture of the Czech Republic.

## Results

### Development of *Porcisia* in *Culicoides sonorensis*

Engorged *C. sonorensis* were dissected on days 1, 3, 6 and 10 PBM. In total, 115 females were analysed. On day 1, the infection rates were 69% (9 positive from 13 gut smears examined) for *P. deanei* and 45% (9 positive from 20 gut smears) for *P. hertigi*. Post defecation, *P. hertigi* infections were lost (we did not detect any positive flies out of 50 females dissected at various time intervals PBM). *Porcisia deanei* promastigotes survived in three females out of 39 dissected; they were detected twice on day 6 PBM and once on day 10 PBM. All infections were localized exclusively in Malpighian tubules (MT) and parasite numbers were moderate (several hundred per female). The representation of metacyclic forms increased with time post bloodmeal, accounting for 33% in females dissected on day 6 PBM and 71% on day 10 PBM, but the sample of promastigotes measured at this late time interval was limited (**Supplementary File 2, 3**).

### Development of *Porcisia* in *L. migonei*

Female *L. migonei* were dissected on days 1, 4 and 7 PBM [as they would not survive until later control days (Guimarães et al., 2016; Becvar et al., 2021)]. A total of 131 females infected with *P. deanei*, 118 females infected with *P. hertigi* and 136 females infected with *L. infantum* were checked. Control *L. infantum* showed a typical suprapylarian development, resulting in heavy infections and colonization of the stomodeal valve in 87% of infected females on day 7 PBM. The infection rate of *Porcisia* species was not significantly different from the control *L. infantum* on day 1 PBM (P = 0.344, d.f. = 2, Chi-Square = 2.132) but decreased significantly with time after feeding (P = 0.003, d.f. = 2, Chi-Square = 11.770 on day 4 PBM and P < 0.001, d.f. = 2, Chi-Square = 23.500 on day 7 PBM).

*Porcisia deanei* promastigotes were detected in 50% of females on the first day PBM, when the infection was localized in the endoperitrophic space surrounded by the peritrophic matrix, but infection rates decreased to 21% on day 4 PBM and 7% on day 7 PBM. On day 4 PBM, three females had moderate or low-intensity infections localized in MT and the abdominal midgut (AMG) pre-defecation of bloodmeal remnants and 9 females post-defecation had low-intensity infections localized predominantly in MT (out of 9 females, only once were parasites detected in the AMG and the thoracic midgut (TMG)). On day 7 PBM, *P. deanei* was detected in four females, in three of which it was localized in MT in low or moderate numbers (less than 100 or 1000 promastigotes, respectively) and once in AMG, TMG and MT (heavy infection, more than 1000 promastigotes). Metacyclic forms were detected on day 4 post bloodmeal, comprising 14% of the sample (**Supplementary File 3**).

*Porcisia hertigi* caused a 48% infection rate on the first day PBM, which dropped to 13% on day 4 PBM and to zero on day 7 PBM. On day 4 PBM, only seven females were infected, all prior to defecation: one with moderate intensity infection in MT and AMG and the remainder with low intensity infection (5 females) or moderate intensity infection (1 female) in the AMG. Among the few promastigotes available for morphological analysis, metacyclic forms were present (**Supplementary File 3**). On day 7 PBM, no infection was detected in 40 females.

### Development of *Porcisia* in *L. longipalpis*

Female sand flies were dissected on days 1, 4, 7 and 11 post bloodmeal (PBM) and their guts were examined for infection under light microscope. A total of 117 females infected with *P. deanei* and 171 females with *P. hertigi* were dissected. In addition, 117 females infected with *L. infantum* were used as a control group.

On the first day PBM, parasites were localized in the endoperitrophic space. The infection rates of both *Porcisia* species were comparable to the 95% infection rate of *L. infantum*, with 69% and 82% females infected with *P. deanei* and *P. hertigi*, respectively (P = 0.102, d.f. = 2, Chi-Square = 4.562).

**Figure 2:**
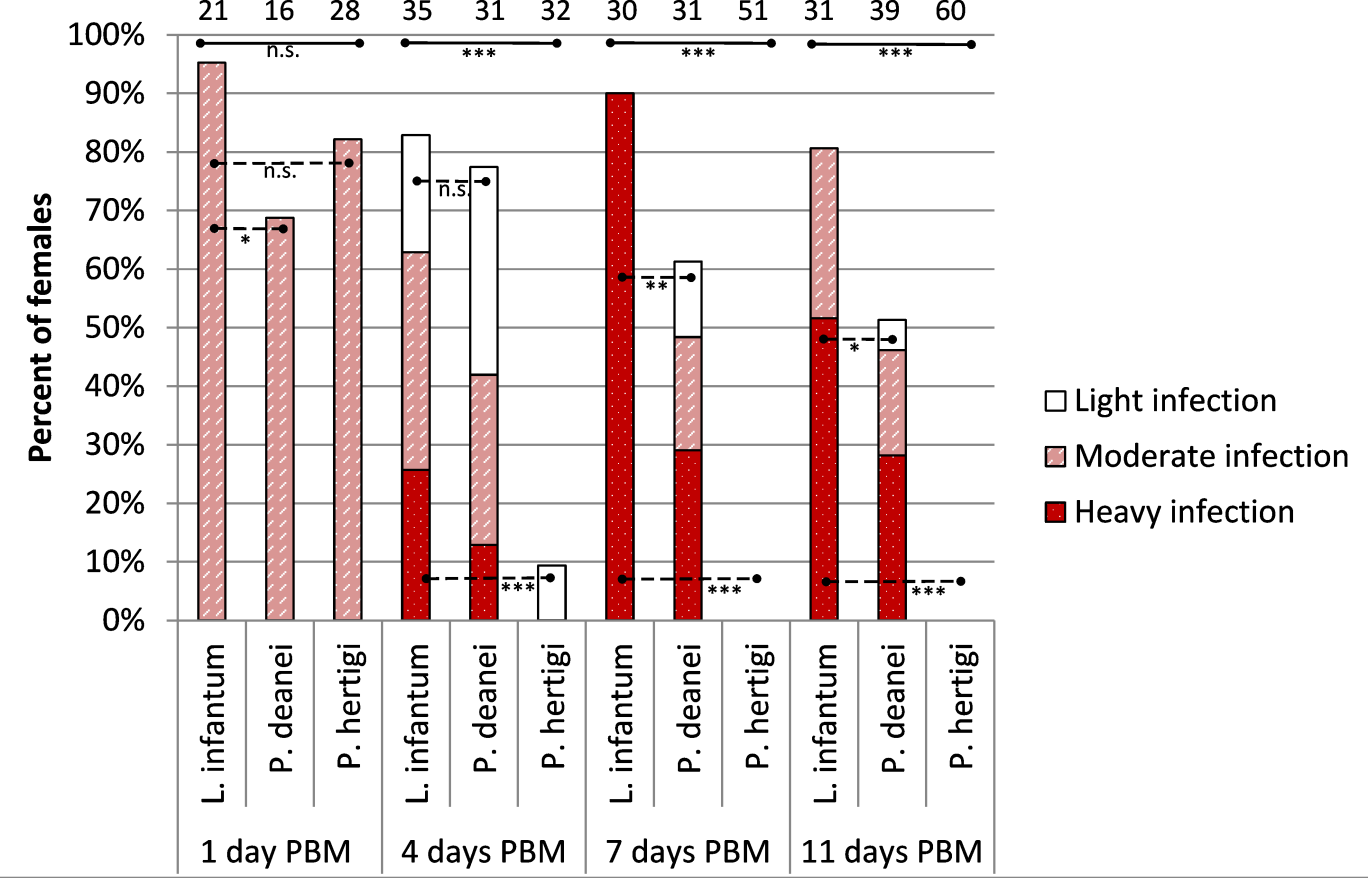
Infection rates and intensity of *P. deanei*, *P. hertigi* and *L. infantum* in *Lutzomyia longipalpis*. Numbers of dissected females are shown above the bars. Significance of between species differences in infection rates were evaluated using Chi-Square test. n.s, P > 0.05; *,P < 0.05; **P < 0.01; ***P < 0.001. Full lines, difference among all three tested species; dashed lines, difference between two interconnected species.

**Figure 3:**
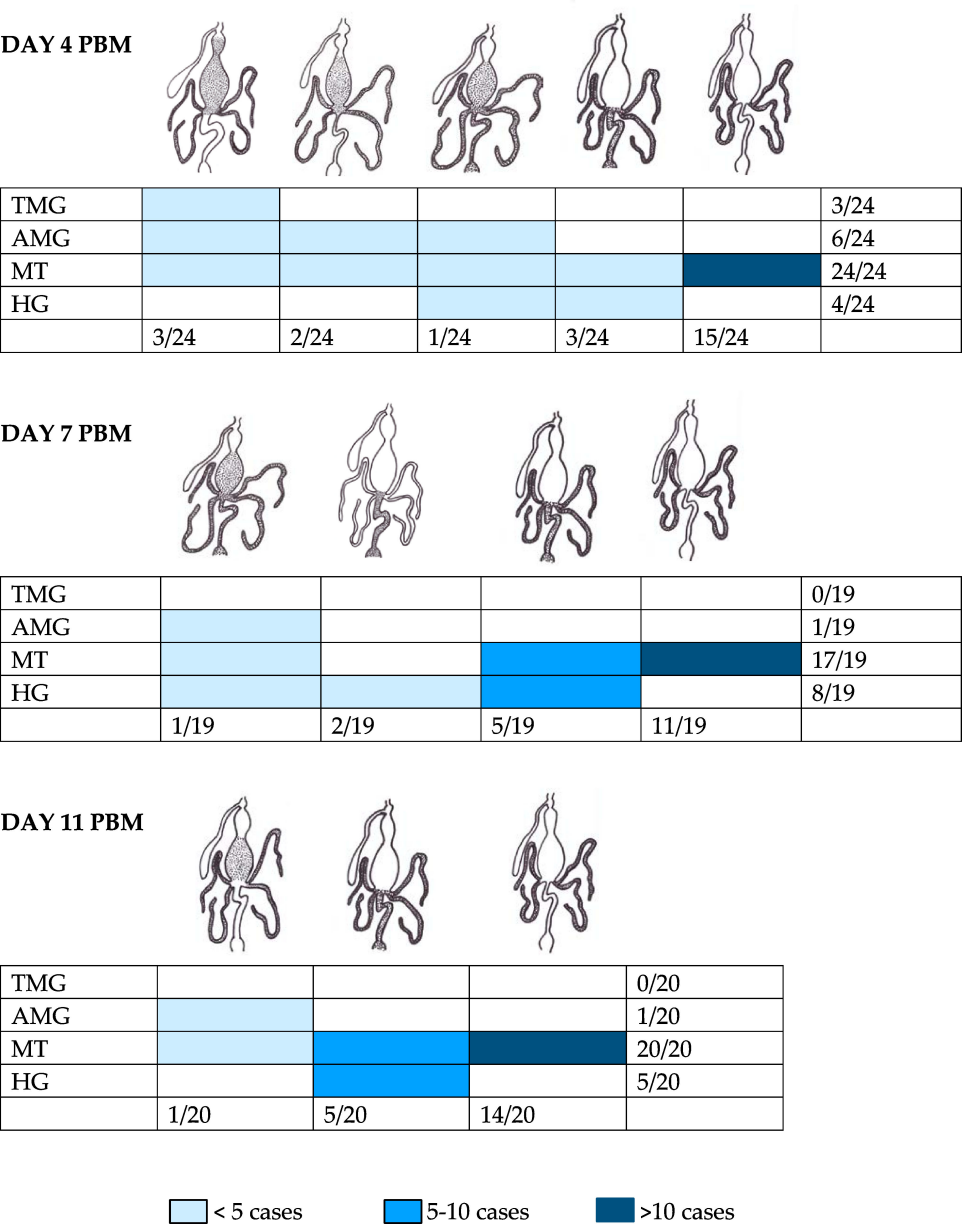
Localization of *Porcisia deanei* in infected *Lutzomyia longipalpis.* TMG, thoracic midgut; AMG, abdominal midgut; MT, Malpighian tubules; HG, hindgut.

On the fourth day PBM, the infection rate and intensity of *P. deanei* infection did not differ significantly from those of *L. infantum*, (P = 0.579, d.f. = 1, Chi-Square = 0.307) while the infection rate of *P. hertigi* was significantly lower (P < 0.001, d.f. = 1, Chi-Square = 36.177), falling below 10% (**Figure 2**). *Leishmania infantum* promastigotes were predominantly localized in the cardia (59%), less frequently remaining restricted to the AMG (10%) or the beginning of the TMG (17%) and in 14% of females, the stomodeal valve (SV) was already colonized. The localization of *P. deanei* infection was quite different (**Figure 3**): in all infected females, MT were infected, either as the only tissue (62% of females) or in combination with AMG and TMG (13%), AMG (8%), AMG and hindgut (HG, 4%) or HG (13%). Midgut infections were mostly light or moderate, while most parasites were always packed in MT.

**Figure 4:**
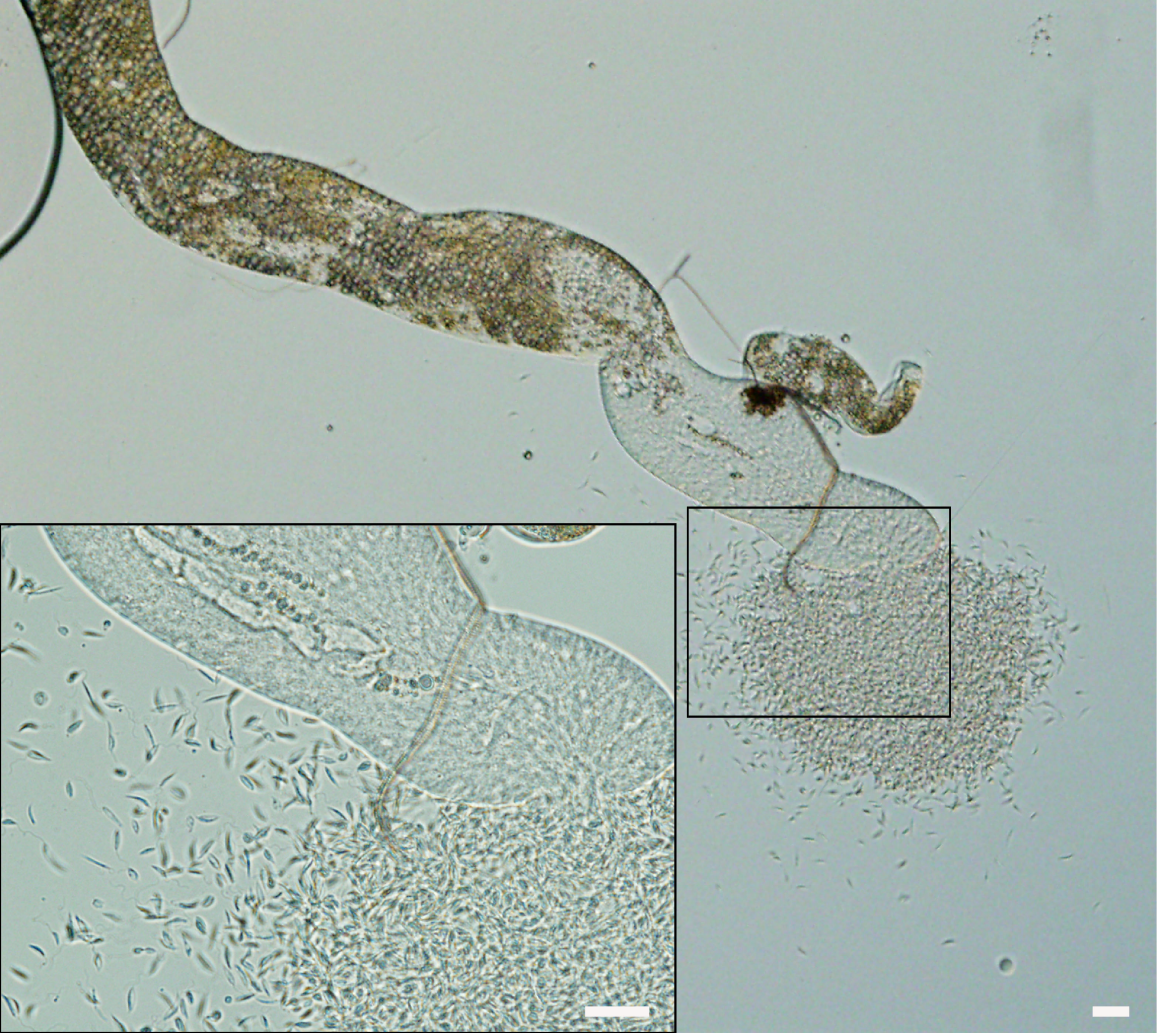
*Porcisia deanei* colonizing Malpighian tubules of *L. longipalpis* on day 7 PBM. Scale bars indicate 20 µm.

In the following days PBM (days 7 and 11), the infection rates of *L. infantum* remained above 80%, infections were mostly heavy and the SV was colonized in all infected females (**Figure 2**). *Porcisia hertigi* was not detected in any dissected female. In contrast, promastigotes of *Porcisia deanei* were observed in 61% and 51% of females on day 7 and 11 PBM, respectively, and nearly 30% were heavily infected. In the majority of females, parasites remained localized only in MT (**Figures 3**, **4**), less frequently and in smaller numbers they were also present in the AMG and/or HG.

### Morphological forms present in *L. longipalpis*

**Figure 5:**
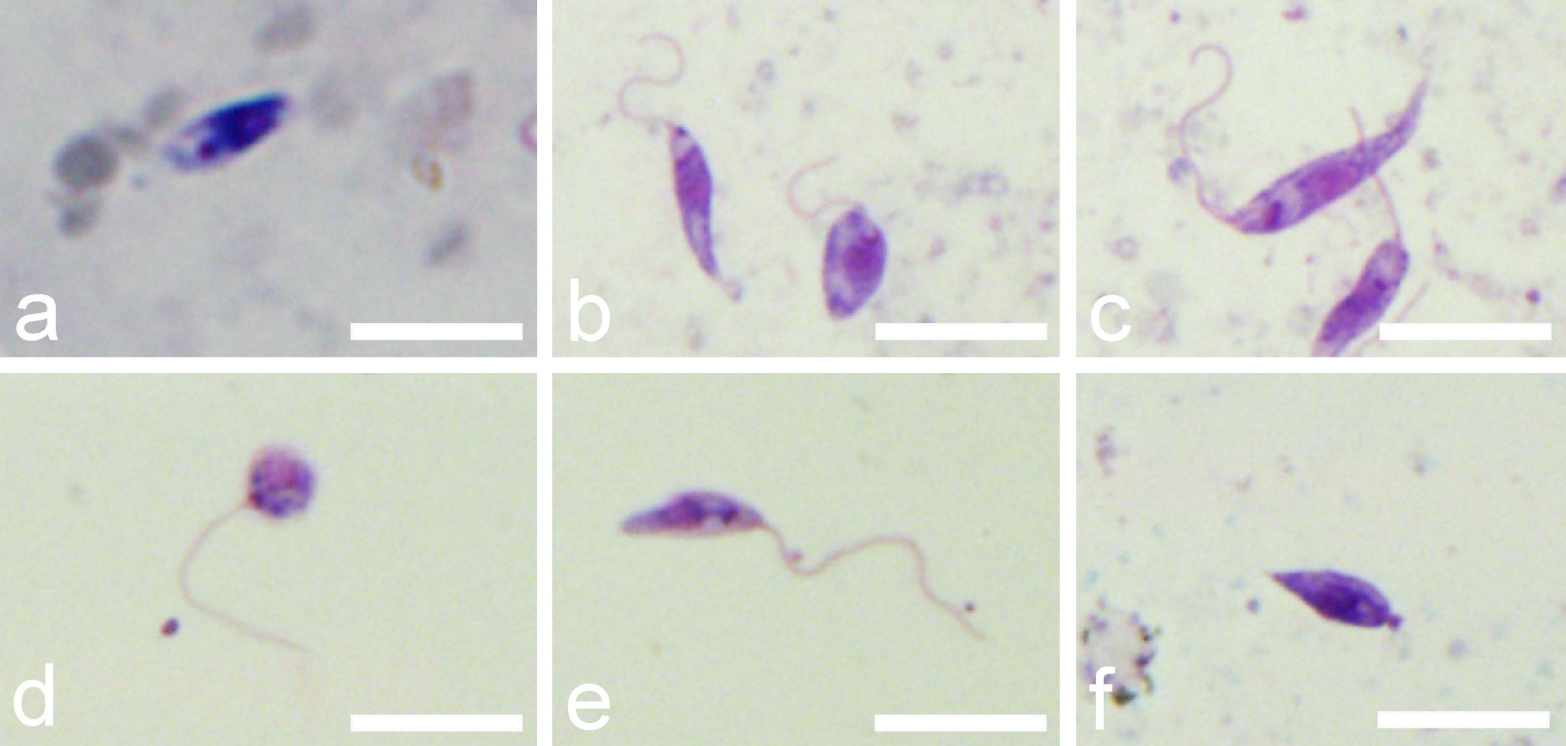
Morphological forms of *P. deanei* present in gut smears of infected *L. longipalpis*. **(A)**, procyclic promastigote; **(B)**, leptomonad forms; **(C)**, elongated nectomonad; **(D)**, round metacyclic form; **(E)**, slender metacyclic form; **(F)**, haptomonad. Scale bars indicate 10 µm.

According to criteria described in Material and Methods, four morphological forms were distinguished on gut smears of infected *L. longipalpis*: procyclic promastigotes, elongated nectomonads, leptomonads and metacyclic forms. Representatives of each form are shown in **Figure 5** and their measurements are provided in **Table 1**.

**Table 1:** Measurements of morphological forms from gut smears of *L. longipalpis*.

Parasite sp.	Day PBM	Morphological form	No. of measurements	Body length		Body width		Flagellar length	
				Mean ± S.D.(µm)	Min-Max	Mean ± S.D.(µm)	Min-Max	Mean ± S.D.(µm)	Min-Max
*L. infantum*	0*	EN	73	16.2 ± 1.7	14-21.1	2.1 ± 0.3	1.2-2.9	16.0 ± 4.8	6.9-30
		LE	121	10.0 ± 2.6	4.7-13.9	2.1 ± 0.5	1.1-4.6	13.5 ± 4.7	4.4-26.8
		MC	5	8.4 ± 0.3	8.0-9.1	2.1 ± 0.3	1.7-2.4	19.3 ± 2.8	16.2-23.5
	1	PP	94	8.5 ± 2.1	4.4-15.2	3.0 ± 1.1	1.2-5.9	1.8 ± 2.9	0.0-11.2
		LE	5	11.0 ± 2.1	8.3-12.6	4.0 ± 0.5	3.5-4.7	13.0 ± 2.9	8.4-16.1
	4	EN	192	20.8 ± 3.2	14.1-32.8	1.4 ± 0.3	0.8-3.2	18.5 ± 3.9	3.4-31.5
		LE	8	12.0 ± 1.0	10.2-13.2	2.1 ± 0.8	1.2-3.8	19.0 ± 3.2	14.2-23.4
	7	EN	154	18.9 ± 2.8	14.0-26.0	2.1 ± 0.6	1.2-4.4	18.3 ± 5.6	1.7-32.4
		LE	33	12.2 ± 1.5	6.9-13.9	2.3 ± 1.0	1.1-5.1	13.3 ± 6.1	1.9-22.8
		MC	3	10.7 ± 0.7	10.0-11.5	3.5 ± 1.5	2.3-5.2	26.9 ± 4.2	22.8-31.3
	11	EN	139	17.6 ± 2.4	14.0-25.9	2.4 ± 0.7	1.1-4.0	17.2 ± 4.8	1.3-29.4
		LE	73	10.5 ± 2.6	5.2-13.9	2.5 ± 0.8	1.3-6.9	12.4 ± 5.3	2.4-24.7
		MC	1	8.3		2.4		20.7	
*P. daenei*	0	EN	153	11.1 ± 2.1	8.0-17.6	2.1 ± 0.4	1.2-3.2	15.2 ± 3.7	6.1-27.3
		LE	34	6.8 ± 0.7	5.4-7.9	2.0 ± 0.3	1.3-2.7	13.4 ± 4.4	3.5-24.5
		MC	13	10.1 ± 1.7	8.1-13.1	2.0 ± 0.5	1.5-3.3	22.1 ± 3.5	17.2-27.1
	1	PP	87	7.7 ± 1.5	4.7-12.8	2.9 ± 0.9	1.6-7.4	0.3 ± 0.8	0.0-3.8
	4	EN	114	12.4 ± 2.2	8.1-17.6	2.0 ± 0.6	1.1-4.9	17.1 ± 4.6	2.0-30.2
		LE	26	6.1 ± 1.3	2.7-7.7	3.3 ± 1.1	1.8-5.1	18.9 ± 8.1	3.0-35.7
		MC	36	10.5 ± 1.8	8.1-14.5	2.1 ± 0.5	1.1-3.2	25.5 ± 4.4	16.9-34.6
	7	EN	98	9.9 ± 1.4	8.0-14.0	2.8 ± 0.8	1.3-5.5	13.0 ± 4.5	0.6-21.3
		LE	67	6.8 ± 0.9	4.4-7.9	3.2 ± 1.1	1.5-8.2	11.6 ± 5.2	1.1-25.0
		MC	14	9.3 ± 1.1	8.0-11.4	3.0 ± 1.0	1.7-5.1	21.3 ± 3.0	17.2-25.9
	11	EN	89	10.7 ± 2.2	8.1-19.4	2.0 ± 0.6	1.0-3.8	12.4 ± 4.2	2.0-21.1
		LE	77	6.3 ± 1.0	4.0-7.8	2.5 ± 0.7	1.3-4.2	10.5 ± 3.6	0.8-20.4
		MC	7	9.3 ± 1.2	8.2-11.8	2.2 ± 0.5	1.2-2.8	20.5 ± 1.8	18.1-23.7
*P. hertigi*	0	EN	124	9.5 ± 1.0	8.0-12.2	2.1 ± 0.4	1.4-3.5	14.2 ± 2.5	8.5-23.0
		LE	67	6.7 ± 1.1	4.2-7.9	2.0 ± 0.3	1.3-2.8	13.3 ± 3.6	7.3-24.9
		MC	8	8.6 ± 0.4	8.2-9.1	2.2 ± 0.5	1.7-2.8	19.0 ± 2.1	16.9-22.7
	1	PP	41	6.1 ± 1.1	3.6-9.2	3.0 ± 0.6	1.9-4.4	0.1 ± 0.2	0.0-1.4

EN, elongated nectomonads; LE, leptomonads; MC metacyclic forms; PP, procyclic forms. *, Day 0 PBM = culture forms used for the experimental infection.

**Figure 6:**
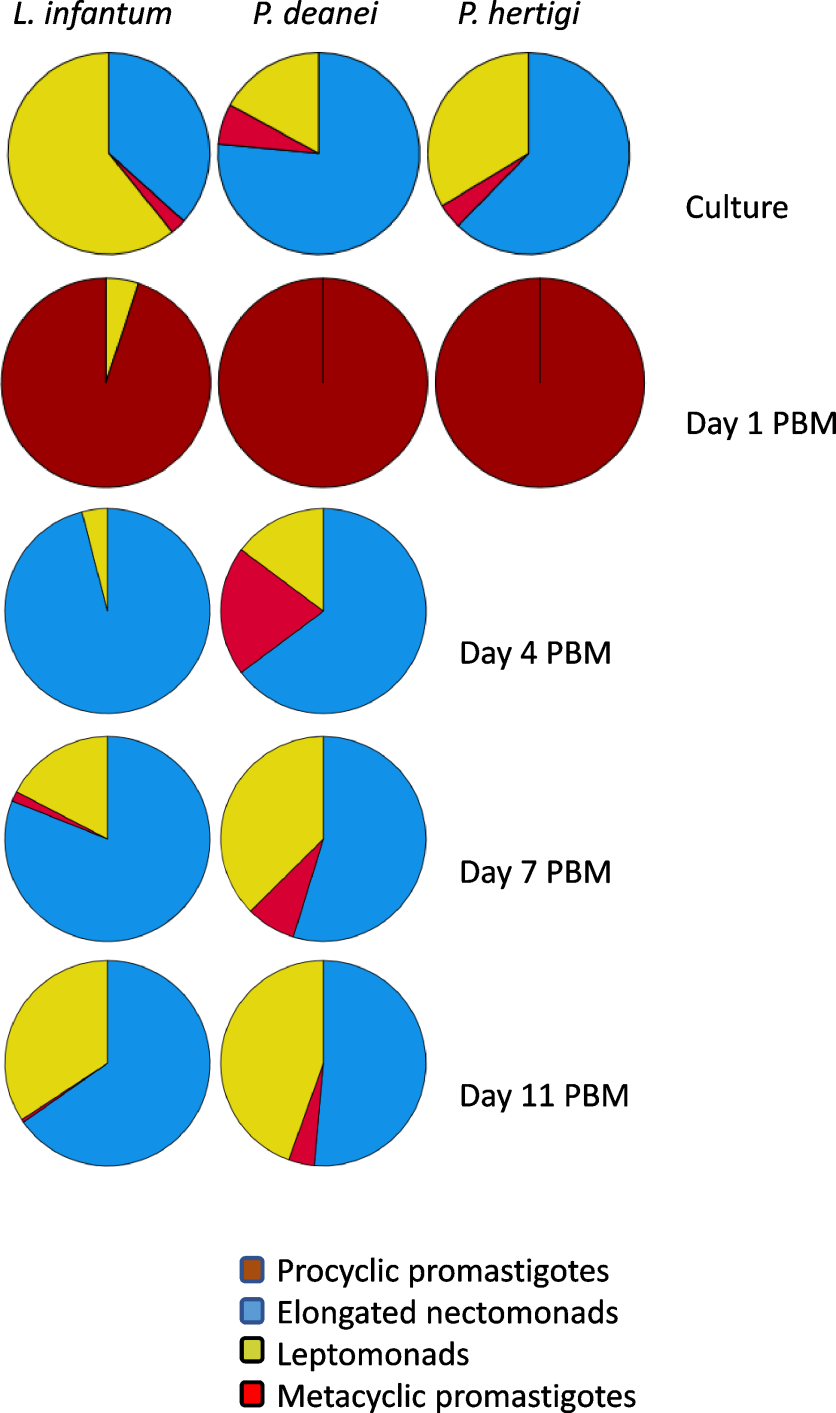
Representation of morphological forms on sand fly guts smears. Haptomonads were not quantified because these attached forms are underscored on gut smears. The numbers of promastigotes can be found in **Table 1**.

Elongated nectomonads prevailed in the *P. deanei* culture used for experimental infection of sand flies (76.5%), leptomonads and metacyclic forms were less represented (17% and 6.5%, respectively). In *P. hertigi*, nectomonads also prevailed (62%) while in *L. infantum*, the predominant forms were leptomonads (61%) (**Figure 6**).

First day PBM, when the infection was restricted to the endoperitrophic space, the population of *P. deanei* homogenously consisted of small procyclic promastigotes with very short or unapparent flagellum (resembling amastigotes, **Figure 5A**). These procyclic forms were sometimes seen in dividing rosettes. *Porcisia deanei* showed the same morphology while in procyclic forms of *L. infantum*, the flagellum was mostly apparent. In *L. infantum*, also forms with the flagellum longer than the body length (leptomonads) appeared in 5%.

On day 4 PBM, nectomonads comprised 64%, leptomonads 15% and metacyclics 20% of *P. deanei* population while in *L. infantum*; nectomonads highly prevailed over leptomonads (96%). In following days PBM, representation of nectomonads decreased in both species while representation of leptomonads increased. Two forms of metacyclics were seen in *P. deanei* – round and slender (**Figures 5D, E**) and in these late infections, metacyclics were more represented in *P. deanei* compare to *L. infantum* (**Figure 6**).

On days 7 and 11 PBM, also several haptomonads were found. These forms were not included to the analysis as these attached forms are principally underestimated on gut smears.

### Presence of *P. deanei* in urine of *L. longipalpis*

**Figure 7:**
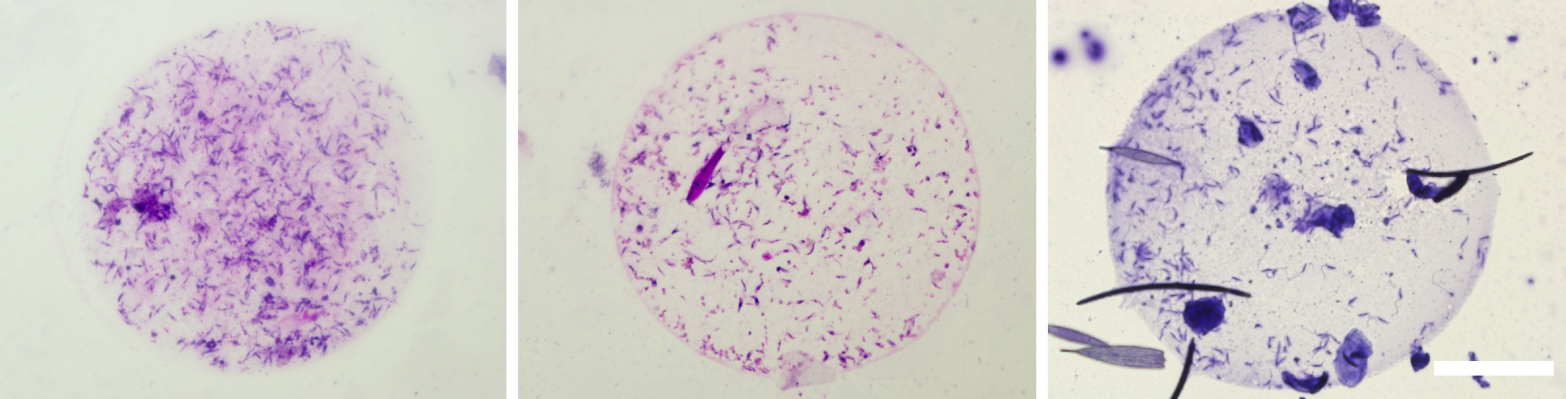
*Porcisia deanei* promastigotes in urine droplets of *Lutzomyia longipalpis*. Scale bar indicates 100 µm.

Microcapillary feeding (Hertig and Mcconnell, 1963) was used for testing of parasite presence in the urine droplets discharged with prediuresis during the feeding of female sand flies 10 - 14 days post infective bloodmeal. Engorged flies were dissected immediately after the experiment to confirm *Porcisia* infections by light microscopy and droplets produced by uninfected females were not further analysed. From five females with *P. daenei* infection confirmed by microscopy, 3 females discharged parasites in urine droplets. They were present in each droplet produced, in numbers ranging from 40 to 350 parasites per droplet. Among discharged parasites, metacyclic forms represented 20-29 percent (**Table 2**; **Figure 7**).

**Table 2:** Numbers of *Porcisia deanei* in urine droplets of *Lutzomyia longipalpis*.

Female	No of droplets	No of parasites per droplet	No (%) of MC forms
1	1	150	40 (27)
2	9	60, 40, 250, 160, 35, 55,40, 270, 350	15(25), 10(25), 50 (20), 40 (25), 10 (29), 20 (36), 10 (25), 70 (26), 100 (29)
3	2	200, 95	40 (20), 20 (21)

### Transmission of *P. deanei* to BALB/c mice

**Figure 8:**
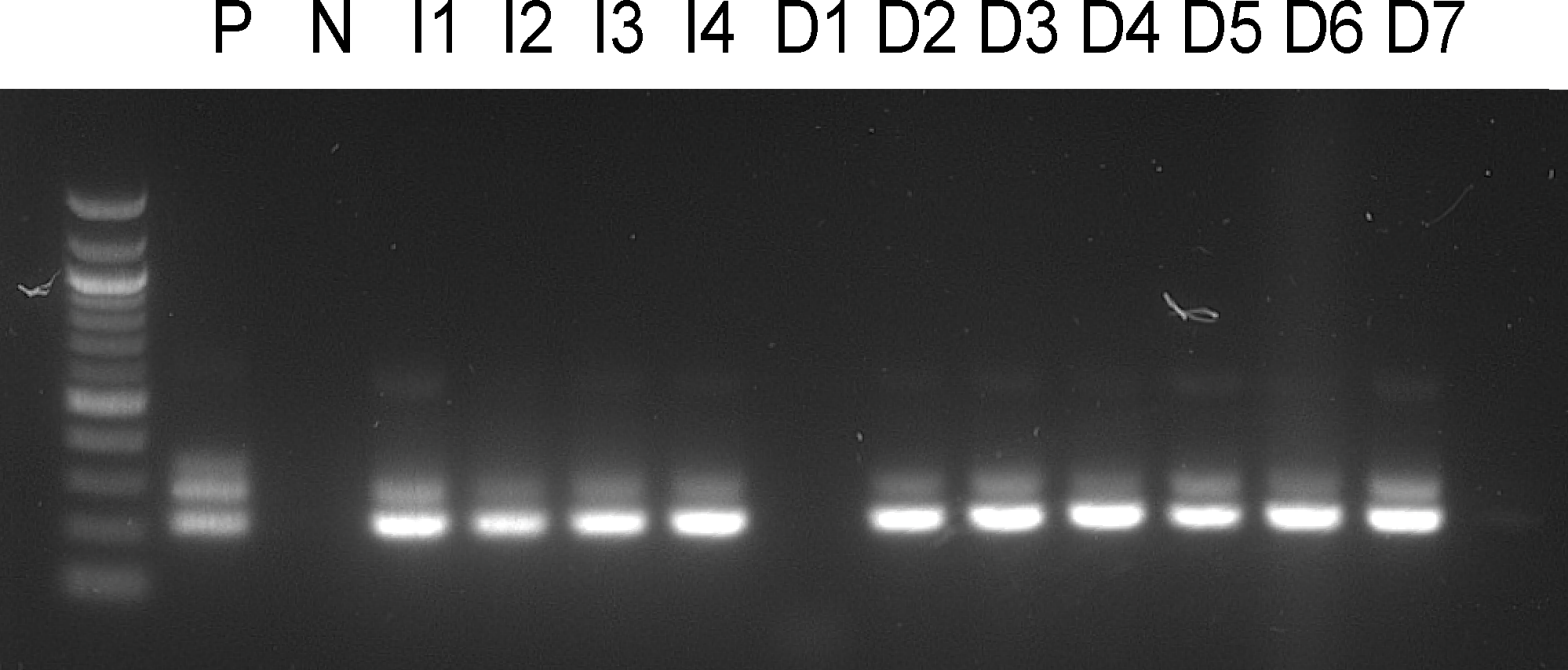
Amplification of a 226 bp *Leishmania* 18S sequence. P, positive control from cultured parasites; N, negative control; I1-4, mice ears exposed to *L. longipalpis* infected with *L. infantum*, mice killed immediately post experiment; D1-3, mice ears exposed to *L. longipalpis* infected with *P. deanei*, mice killed immediately post experiment; D4-5, mice ears exposed to *L. longipalpis* infected with *P. deanei* and D6-7, their draining lymph nodes, mice killed 4 days post experiment.

To prove the transmission potential of *L. longipalpis* infected with *P. deanei*, females 10 days post infective blood meal were allowed to feed on the ear pinnae of anaesthetized BALB/c mice. As a control, sand flies infected with *L. infantum* were used. Numbers of engorged infected females are summarized in **Table 3**. For detection of parasites in mice tissues, a nested PCR assay with primers flanking a 226 bp 18S sequence was used. The presence of *Leishmania* DNA was confirmed in 2/3 ears and all 4 ears of mice exposed to *L. longipalpis* infected with *P. deanei* and *L. infantum*, respectively, sacrificed immediately post exposition. In a following experiment, the mouse exposed to sand flies infected with *P. deanei* was sacrificed later, 4 days post experiment, and also ear draining lymph nodes were sampled. Parasites were detected in both ears and both ear draining lymph nodes (**Table 3**; **Figure 8**).

**Table 3:** Microscopical examination of *L. longipalpis* females allowed to feed on BALB/c mice for transmission of parasites and result of PCR detection of *Leishmania* minicircle kDNA in mouse ears.

Parasite species	Sample (mouse ear)	No. ofengorged females	No. of infected engorged females	Sampling post exposure	Transmission confirmed by PCR
*P. deanei*	D1	15	8	Immediately	No
	D2	14	5	Immediately	Yes
	D3	16	7	Immediately	Yes
*L. infantum*	I1	9	3	Immediately	Yes
	I2	17	9	Immediately	Yes
	I3	13	8	Immediately	Yes
	I4	12	7	Immediately	Yes
*P. deanei*	D4	8	3	4 days	Yes
	D5	2	2	4 days	Yes

## Discussion

While the biology of the genus *Leishmania* with its many medically important species has been extensively studied, knowledge of the life cycle of the genus *Porcisi*a remains poor. These flagellates are apparently harmless to their hosts, the Neotropical porcupines, although they are found in the skin throughout the body and also visceralize (Herrer, 1971). They are highly host specific and well adapted to porcupines and are capable of inducing extremely high infection rates in host populations (Herrer et al., 1966; Lainson and Shaw, 1977). However, the vectors of *Porcisia* remained a mystery.

In nature, *Porcisia* DNA was detected by nested PCR in *Lutzomyia antunesi* in Brazil (Thies et al., 2018). However, this finding does not imply that *L. antunesi* is a competent vector. Only microscopical observation can document the localization of infection and the time post bloodmeal, and evidence that the parasites survived a non-specific early phase of the infection that may be lost with defecation of blood remnants.

Laboratory experimental infections of various sand fly species with *Porcisia* have not yet demonstrated a vectorial role for sand flies. Lainson et al. (1977) described the poor development of *P. deanei* and *P. hertigi* in *L. longipalpis*, but their study examined the hindgut development of 14 New World’s *Leishmania* species and only 2 and 9 female *L. longipalpis* were infected with *P. hertigi* and *P. deanei*, respectively. After an initial period of low multiplication in the midgut, the sparse parasites migrated to the hindgut and MT where they were observed to be free swimming without attachment to any part of the gut wall (Lainson et al., 1977). Similarly, Croft and Molyneux (1979) reported a low infection rate of 10% (5/50) for *L. longipalpis*. Infections were weak and consisted of a few promastigotes in the posterior and anterior midgut, pylorus and MT (Croft and Molyneux, 1979). Very weak development of *P. hertigi* has also been reported in the local sand fly species *Lutzomyia sanguinaria* (Herrer et al., 1966) and *Lu. gomezi* (Lainson and Shaw, 1977).

In our experiments, *P. hertigi* failed to form mature infections in all three vector species: *L. migonei, L. longipalpis* and *C sonorensis* while *P. deanei* survived defecation in all three tested vectors. However, while *P. deanei* only infected a low percentage of *L. migonei* and *C. sonorensis* females, it caused heavy infections in *L. longipalpis* females, localized predominantly in MT with the presence of a high percentage of metacyclic forms.

The morphology of *Porcisia* parasites has so far been thoroughly described only in cells from culture and vertebrate hosts. Amastigotes of *P. deanei* have been reported to be considerably large, averaging 6.1 x 3.1 - 3.7 µm with an elongated kinetoplast (Deane et al., 1974; Lainson and Shaw, 1977) whereas amastigotes of *L. hertigi* are smaller, 3.5 - 4.8 x 1.2 - 2.5 µm (Herrer, 1971) with a round kinetoplast (Herrer, 1971). Promastigotes in culture showed a diversity of short and elongate forms in both species, measuring 6.0 - 18.0 x 1.5 - 2.5 µm with a flagellum of 7 - 24 µm in *P. deanei* (Lainson and Shaw, 1977) and 7.5 - 20.5 x 1.3 - 4.1 µm with a flagellum of 9.1 - 35.5 µm in *P. hertigi* (Herrer, 1971). In our study, the promastigotes of *P. deanei* in culture were larger (5.4 - 17.6 x 1.2 – 3.3 µm) than those of *P. hertigi* (4.2 - 12.2 x 1.3 - 2.8 µm), and the same was true for procyclic promastigotes in *L. longipalpis* on the first day PBM with 4.7 - 12.8 x 1.6 - 7.4 µm in *P. deanei* compared to 3.6 - 9.2 x 1.9 – 4.4 µm in *P. hertigi*. Procyclic promastigotes developing within the peritrophic matrix were characterized by very short or even invisible flagella in both *Porcisia* species (average 2.9 µm in *P. deanei* and 3.0 µm in *P. hertigi*) and resembled sphaeromastigotes or amastigotes reported as the first *Trypanosoma cruzi* stages present in the bloodmeal of reduviid bugs (Tyler and Engman, 2001). Post defecation of *L. longipalpis* females, *P. deanei* developed late infections with a persistent dominance of elongated nectomonads (8.0 - 19.4 x 1.0 - 5.5 µm) and a lower representation of short leptomonads (2.7 – 7.9 x 1.3 – 8.2 µm). However, it should be noted that division of promastigotes into nectomonads and leptomonads according to their body length is arbitrary (see **Supplementary File 1**). Importantly, metacyclic forms with an ellipsoid or rounded body shape and flagellum at least twice the body length accounted for a significant proportion of promastigotes in late infections, whereas haptomonads were very rarely found on gut smears and were never observed *in vivo*. Similarly, Lainson et al. (1977) did not find attached forms of *P. deanei* on any occasion on any part of the gut wall of *L. longipalpis*.

The predominant localization of promastigotes in MT was surprising because MT are rarely used during the development of both monoxenous and dixenous trypanosomatids in their vectors. MT infections account for only 2% of infections of monoxenous trypanosomatids in Heteropteran and Dipteran hosts, being combined with parasite presence in the adjacent mid and/or hindgut (Lukeš et al., 2018). In the dixenous *Leishmania* genus, three types of development have been recognized: suprapylarian development, typical of the subgenus *Leishmania*, is restricted to the mesenteron; peripylarian development, known in the subgenus *Viannia* and some *Sauroleishmania* species, also involves multiplication in the hindgut prior to the anterior migration and colonization of the stomodeal valve; and finally, hypopylarian development in other *Sauroleishmania* species is restricted to the hind gut (Lainson and Shaw, 1987). Occurrence of low numbers of *Leishmania* promastigotes in MT, reported in natural or experimental infections, has been interpreted as a consequence of peristaltic backwash from the midgut (Lainson and Shaw, 1968) and invasion of MT by *Leishmania* has been more frequently observed in unnatural vector- parasite pairs or atypical experimental conditions like maltose or fructose diet and reduced temperature (Rangel et al., 1985; Walters et al., 1987; Anez et al., 1989; Nieves and Pimenta, 2000; Ticha et al., 2021). In the dixenous genus *Endotrypanum*, which is closely relative to *Porcisia*, development in MT has been observed more frequently than in *Leishmania*, but in variable proportion in different sand fly species and in a lower percentage of females compared with midgut infections (Shaw, 1981; Franco et al., 1997). The type of development observed in this study - colonization of MT as the dominant body part of the vector, was not described yet.

We sought to answer the important question of whether this curious localization is an artefact resulting from the fact that *L. longipalpis* is not a natural vector of *P. deanei*, or whether this sand fly species can serve as a competent vector and the infection can be transmitted to vertebrates. Malpighian tubules are the major osmoregulatory and excretory organs of insects, responsible for the production of isosmotic filtrate from the hemolymph, the primary urine that carries excretory products of metabolism and toxic compounds into the hindgut (Nocelli et al., 2016). Sand flies begin urine production immediately upon feeding on the host to concentrate the proteins of the bloodmeal and restore weight and water balance (Sádlová et al., 1998). During this process, called prediuresis, parasites may be excreted in urine droplets, allowing for contaminative transmission, as demonstrated in a model of *Phlebotomus duboscqi* infected with *L. major* (Sádlová and Volf, 1999). Therefore, free-swimming *Porcisia* promastigotes can theoretically be passed from MT of *L. longipalpis* to the hindgut and reach the host skin in urine droplets during prediuresis. To prove this theory, we inspected urine droplets of infected *L. longipalpis* exposed to capillary feeding and observed the droplets packed with hundreds of parasites with 20-29 percent of metacyclics.

This finding encouraged us to perform the following step, transmission to the mammalian hosts. Female *L. longipalpis* infected with *P. deanei* were allowed to feed on ears of anaesthetized BALB/c mice and PCR confirmed successful transmission - DNA of *P. deanei* was detected in the ear tissue not only immediately post feeding but also 4 days post experiment, in samples from both the ear and the ear draining lymph node. These results demonstrated that *L. longipalpis* is a competent vector of *P. deanei*, but the involvement of other phlebotomine species in its transmission is not excluded. In *C. sonorensis*, although surviving in a small percentage of females, *P. deanei* formed heavy infections containing metacyclic stages, also localized in the MT. Therefore, the search for Neotropical vectors of *Porcisia* parasites may also involve biting midges.

Contaminative transmission is rather rare in trypanosomatids. Transmission by excretion of metacyclic forms into the reduviid bite wound is typical for *Trypanosoma cruzi* (Tyler and Engman, 2001). In *Leishmania*, the predominant route by which promastigotes enter the host is certainly the transmission by bite. Contaminative transmission by ingestion of infected female sand flies presumably takes place in the subgenus *Sauroleishmania* (Lainson and Shaw, 1987) and may occur in transmission of *Leishmania* to dogs while in man, rather little importance is attributed to occasional contaminative transmission by crushing the fly on the skin as it bites (reviewed by (Killick-Kendrick, 1979). However, the frequent occurrence of live *L. major* have been confirmed in the prediuretic liquid of *P. duboscqi* and transmission of metacyclic forms by mosquito prediuretic fluid has also been considered for mammalian *T. congolense* (Mutero and Mutinga, 1993) and avian *T. theileri* (Brotánková et al., 2022). Contaminative transmission is not as conditioned by the number or localization of parasites in the gut of the vector like as is the case for transmission by bite (Sádlová and Volf, 1999). Therefore, delivery of infective forms to the host in urine may be a primitive mode of transmission to which *Porcisia* adapted by its unique localization in vector´s Malpighian tubules.

## Data availability statement

The raw data supporting the conclusions of this article will be made available by the authors, without undue reservation.

## Ethics statement

The animal study was reviewed and approved by The Committee on the Ethics of Laboratory Experiments of Charles University in Prague.

## Author contributions

Conceptualization, methodology, formal analysis, writing - original draft preparation, JSa; investigation, DB, JSa, TB, and BV; writing—review and editing – PV, JSh, and ME; supervision, JSa, and PV; funding acquisition, PV and JSa. All authors have read and agreed to the published version of the manuscript.

## Funding

JSa and PV were funded by ERD Funds, project CePaViP (https://ec.europa.eu/regional_policy/en/funding/erdf/, grant No. CZ.02.1.01/0.0/0.0/16_019/0000759). Shipment of *Culicoides* was funded by Research Infrastructures for the control of vector-borne diseases (Infravec2, https://infravec2.eu/), which has received funding from the European Union’s Horizon 2020 research and innovation programme under grant agreement No 731060 by JSa. ME is funded by the Defra national *Culicoides* laboratory.

## Acknowledgments

We thank to Lenka Krejcirikova and Kristyna Srstkova for the administrative and technical support.

## Conflict of interest

The authors declare that the research was conducted in the absence of any commercial or financial relationships that could be construed as a potential conflict of interest.

## Publisher’s note

All claims expressed in this article are solely those of the authors and do not necessarily represent those of their affiliated organizations, or those of the publisher, the editors and the reviewers. Any product that may be evaluated in this article, or claim that may be made by its manufacturer, is not guaranteed or endorsed by the publisher.
