# Supplementary material for: Porcisia transmission by prediuresis of sand flies
Source: Front Cell Infect Microbiol. 2022 Aug 10;12:981071. doi: 10.3389/fcimb.2022.981071 (PMC9399930; doi:10.3389/fcimb.2022.981071)

Suppl. file 2. Morphological forms of *P. deanei* and *P. hertigi* present in gut smears of infected *Culicoides sonorensis*.

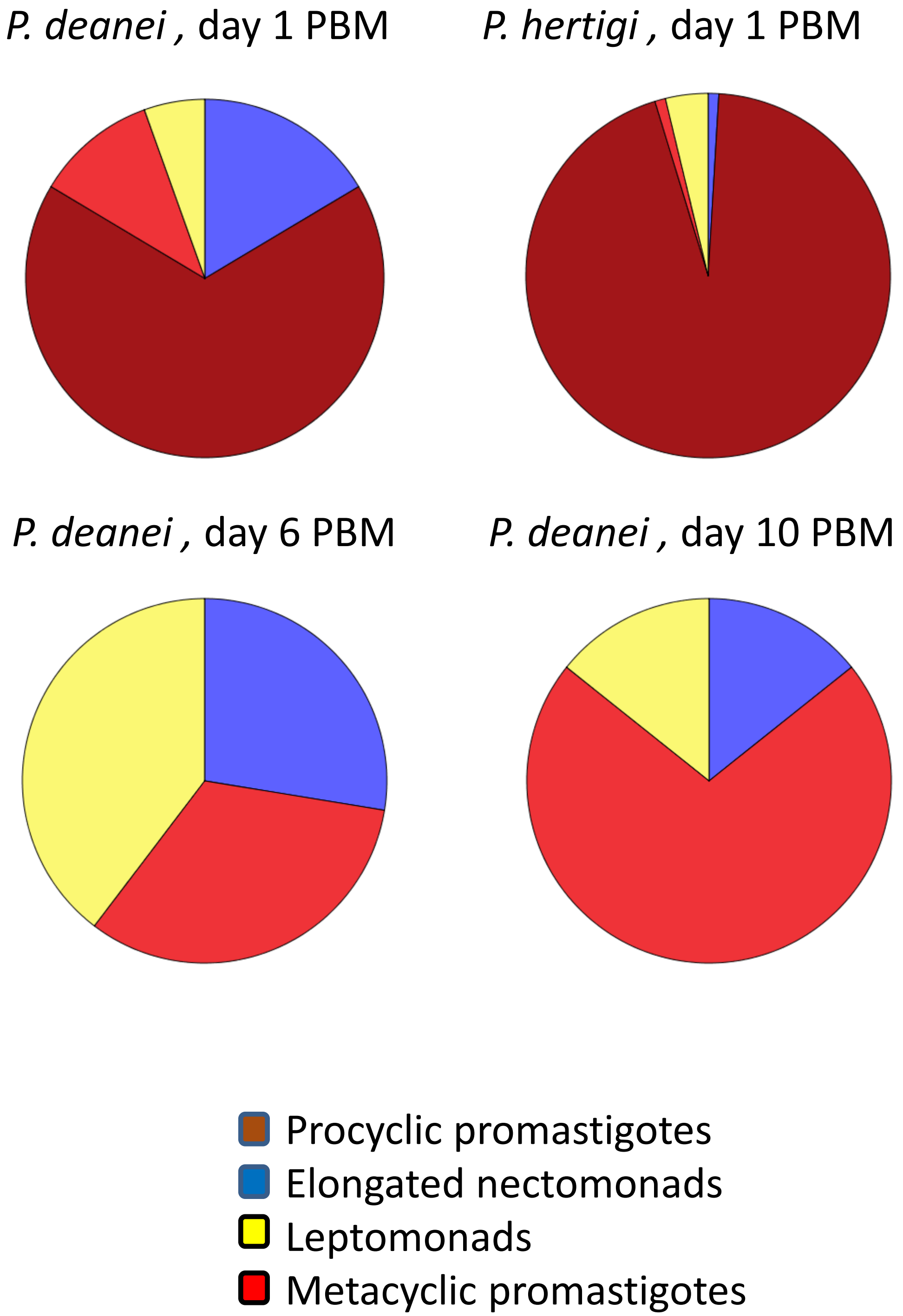

Supplement: Supplementary file 1 [file DataSheet_1.pdf]
