# Supplementary material for: Porcisia transmission by prediuresis of sand flies
Source: Front Cell Infect Microbiol. 2022 Aug 10;12:981071. doi: 10.3389/fcimb.2022.981071 (PMC9399930; doi:10.3389/fcimb.2022.981071)
Supplement: Supplementary file 2 [file Table_1.doc]

Body length of promastigotes of L. infantum, P. deanei and P. hertigi measured on the whole sample of promastigotes from the culture and sand fly guts. Yellow arrows indicate the threshold between nectomonads and leptomonads, i.e., 14 µm in L. infantum and 8 µm in both Porcisia species.	
			
			
		 L. infantum	P.deanei	P.hertigi	
	N	901	815	240	
	Mean (Std. Error)	15,462 (0,1744)	9,502 (0,0952)	8,079 (0,1174)	
	95% Confidence Interval for Mean	Lower Bound	15,120	9,315	7,848	
		Upper Bound	15,804	9,689	8,310	
	5% Trimmed Mean	15,449	9,422	8,103	
	Median	15,800	9,200	8,300	
	Variance	27,406	7,380	3,306	
	Std. Deviation	5,2350	2,7166	1,8182	
	Minimum	4,4	2,7	3,6	
	Maximum	32,8	19,4	12,2	
	Range	28,4	16,7	8,6	
	Interquartile Range	7,8	3,9	2,5	
	Skewness	-,066 (0,081)	,457 (0,086)	-,305 (0,157)	
	Kurtosis	-,729 (0,163)	-,095 (0,171)	-,471 (0,313)	
