# Supplementary material for: Porcisia transmission by prediuresis of sand flies
Source: Front Cell Infect Microbiol. 2022 Aug 10;12:981071. doi: 10.3389/fcimb.2022.981071 (PMC9399930; doi:10.3389/fcimb.2022.981071)
Supplement: Supplementary file 3 [file Table_2.docx]

Suppl. File 3.

Measurements of morphological forms from gut smears of *Culicoides sonorensis*.

| Parasite  sp. | Day PBM | Morpho-logical form | No. of measure-ments | Body length | | Body width | | Flagellar length | |
| --- | --- | --- | --- | --- | --- | --- | --- | --- | --- |
|  |  |  |  | Mean ± S.D.(µm) | Min-Max | Mean ± S.D.(µm) | Min-Max | Mean ± S.D.(µm) | Min-Max |
| *P. deanei* | 1 | EN | 12 | 9.2±1.0 | 8.0-10.9 | 2.1±0.4 | 1.4-2.9 | 10.2±6.6 | 0.0-18.4 |
|  |  | LE | 4 | 5.9±1.1 | 4.7-7.4 | 1.9±0.1 | 1.9-2.1 | 7.9±2.0 | 6.3-10.7 |
|  |  | MC | 8 | 6.9±1.4 | 4.7-9.3 | 2.0±0.3 | 1.6-2.5 | 16.9±2.0 | 13.7-19.6 |
|  |  | PP | 49 | 6.9±1.4 | 4.8-13.0 | 2.3±0.4 | 1.6-3.1 | 1.3±2.2 | 0.0-6.7 |
|  | 6 | EN | 16 | 9.5±1.0 | 8.1-11.7 | 2.1±0.5 | 1.3-2.9 | 10.6±5.4 | 1.9-17.2 |
|  |  | LE | 23 | 6.3±0.9 | 4.4-7.8 | 2.1±0.5 | 1.2-3.2 | 7.3±4.1 | 0.8-13.6 |
|  |  | MC | 19 | 7.4±1.8 | 4.7-10.7 | 1.9±0.4 | 1.4-2.9 | 16.1±3.3 | 11.4-22.7 |
|  | 10 | EN | 1 | 9.7 |  | 1.6 |  | 2.3 |  |
|  |  | LE | 1 | 7.9 |  | 1.6 |  | 13.4 |  |
|  |  | MC | 5 | 6.7±1.6 | 4.6-8.3 | 2.3±0.2 | 2.0-2.4 | 19.3±3.8 | 13.2-23.6 |
| *P. hertigi* | 1 | EN | 1 | 10.8 |  | 2.8 |  | 12.4 |  |
|  |  | LE | 4 | 6.2±1.1 | 4.9-7.2 | 2.9±0.7 | 2.2-3.9 | 8.9±3.5 | 6.1-13.8 |
|  |  | MC | 1 | 4.3 |  | 1.9 |  | 10.5 |  |
|  |  | PP | 100 | 5.2±1.1 | 2.9-8.7 | 2.4±0.5 | 1.5-4.8 | 0.1±0.5 | 0.0-2.4 |

EN, elongated nectomonads; LE, leptomonads; MC metacyclic forms; PP, procyclic forms.

Measurements of morphological forms from gut smears of *L. migonei*.

| Parasite  sp. | Day PBM | Morpho-logical form | No. of measure-ments | Body length | | Body width | | Flagellar length | |
| --- | --- | --- | --- | --- | --- | --- | --- | --- | --- |
|  |  |  |  | Mean ± S.D.(µm) | Min-Max | Mean ± S.D.(µm) | Min-Max | Mean ± S.D.(µm) | Min-Max |
| *P. deanei* | 4 | PP | 8 | 12.0±1.6 | 9.1-13.5 | 2.1±0.8 | 1.1-3.3 | 7.6±2.8 | 3.6-11.5 |
|  |  | EN | 14 | 11.5±2.6 | 8.4-17.9 | 1.9±0.9 | 1.0-4.4 | 22.9±7.2 | 11.2-34.9 |
|  |  | LE | 2 | 6.9±0.6 | 6.5-7.4 | 2.4±0.4 | 2.1-2.7 | 8.3±1.8 | 7.1-9.6 |
|  |  | MC | 4 | 6.6±1.0 | 5.1-7.38 | 2.0±0.6 | 1.2-2.6 | 17.9±3.9 | 14.8-23.5 |
|  | 7 | EN | 5 | 11.5±1.6 | 10.3-14.2 | 1.7±0.5 | 1.2-2.4 | 18.8±3.8 | 13.0-229 |
|  |  | LE | 1 | 7.9 |  | 2.3 |  | 7.0 |  |
| *P. hertigi* | 4 | LE | 1 | 7.5 |  | 2.3 |  | 8.2 |  |
|  |  | MC | 2 | 6.6±1.6 | 5.5-7.8 | 2.1±0.8 | 1.6-2.7 | 22.7±0.3 | 22.5-23.0 |

EN, elongated nectomonads; LE, leptomonads; MC metacyclic forms; PP, procyclic forms.
